# Gain-of-function mutations in Trim71 linked to congenital hydrocephalus

**DOI:** 10.1371/journal.pbio.3001993

**Published:** 2023-02-09

**Authors:** Yingying Chen, Xianfa Yang, Naihe Jing

**Affiliations:** 1 Guangzhou Laboratory, Guangzhou, China; 2 CAS Key Laboratory of Regenerative Biology, Guangdong Provincial Key Laboratory of Stem Cell and Regenerative Medicine, Guangzhou Institutes of Biomedicine and Health, Chinese Academy of Sciences, Guangzhou, China

## Abstract

Mutations in the RNA-binding domain of Trim71 can cause congenital hydrocephalus (CH). This Primer explores a recent study in PLOS Biology which shows that two known CH-associated Trim71 mutations lead to distinct ectopic RNA binding; these gain-of-function mechanisms influence neurogenesis in vitro and may contribute to CH pathology.

## Teaser

The genetic basis of congenital hydrocephalus is only partially understood. A new study in *PLOS Biology* reports a potential gain-of-function pathological mechanism of congenital hydrocephalus in mouse embryonic stem cells that involves Wnt–β-catenin signaling pathway regulation.

Hydrocephalus, one of the most severe and multifactorial neurological disorders, is classically defined as an active distension of the ventricular system, resulting from inadequate passage of cerebrospinal fluid (CSF) from its point of production in the choroid plexus to its sites of absorption into the systemic circulation [1]. It is estimated to affect one in 1,000 infants and accounts for about 3% of all pediatric hospital charges [2]. Impaired CSF flow, reabsorption, and/or excessive CSF production are classically thought to be the major causes for hydrocephalus. Clinically, patients affected with hydrocephalus are routinely treated with neurosurgical CSF shunting, but neurodevelopmental outcomes are poor and recurrence or morbidity remain common [3]. The lack of effective medical strategies for treating hydrocephalus highlights the importance of a deep understanding of the pathology.

Recent findings from whole-exome sequencing (WES) of patients with congenital hydrocephalus (CH) revealed that genetic mutations contributed to more than 20% of sporadic CH cases [4]. The frequent occurrence of mutations in genes encoding neurogenesis regulators indicates that an aberrant neurodevelopment process may be the pathological cause of human idiopathic CH. However, genetic studies also revealed that heterogeneous mutations could occur in a single CH-related gene. Thus, whether diverse genetic mutations (approximately 100 apparently heterogeneous CH risk genes) could converge to produce a unified molecular mechanism remains largely unexplored. For example, *TRIM71*, which encodes the RNA-binding protein tripartite motif containing 71 (also known as LIN-41), has recently been identified as a CH-causing gene in humans with evidence of pathological mutations at multiple sites [4,5].

Previous mechanistic studies had shown that mouse embryos with genetic knockout of Trim71 displayed a failure in neural tube closure and a lethal phenotype at around embryonic day (E) 9.5, and highlighted the importance of Trim71 in cortical neurogenesis [6]. A mouse model harboring a CH-associated mutation in Trim71 (R595H, homolog of human R608H-TRIM71) showed ventriculomegaly with increased intracranial volume, which phenocopied pathological features of patients with CH [7]. Interestingly, the differences in phenotypes between Trim71-KO mice and those with the CH-associated mutation raised the possibility of heterogeneous pathological mechanisms in CH.

**Fig 1:**
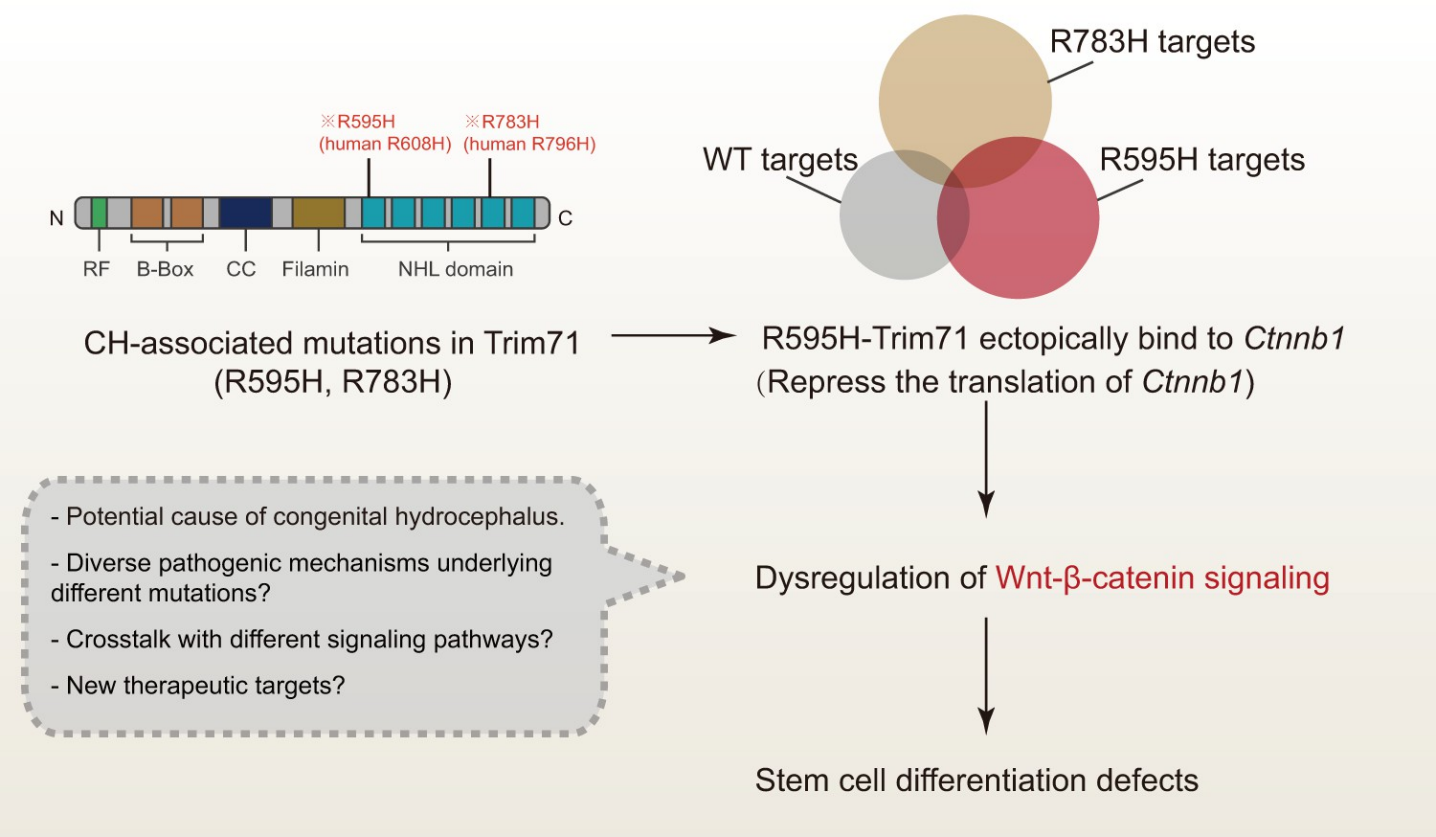
Potential pathological mechanisms of congenital hydrocephalus associated with Trim71 mutations. Mutations discovered in Trim71 (R595H and R783H) are located in the NHL domain. Mutant forms of Trim71 ectopically bind to distinct repertoires of target mRNAs. The ectopic binding to *Ctnnb1* transcripts repressed translation and production of β-catenin. Dysregulation of Wnt–β-catenin signaling may affect stem cell neural differentiation, which could potentially cause congenital hydrocephalus. CC, coiled coil; RF, ring finger; WT, wild-type.

In their new study, Liu and colleagues [8] took advantage of CRISPR-based genome editing and a mouse embryonic stem cell in vitro differentiation system to investigate the effect of 2 different CH-associated mutations (R595H-Trim71 and R783H-Trim71) during the neural differentiation process (Fig 1). Generally, they found that the 2 CH-associated mutations produced similar defects during neural differentiation. This result was in line with published findings using mouse embryos (Trim71-KO and a CH-associated mutant) that highlighted the important role of Trim71 in neurogenesis [6,7,9]. Surprisingly, by using crosslinking immunoprecipitation and sequencing (CLIP-seq), Liu and colleagues [8] provided evidence that these 2 different mutant Trim71 proteins bind to distinct repertoires of target mRNAs via gain-of-function mechanisms. In particular, R595H-Trim71 ectopically binds to *Ctnnb1* transcripts, encoding β-catenin, and thus represses their normal translation. Deficiency in neural differentiation of R595H-Trim71 cells could be alleviated through modulating Wnt–β-catenin signaling. This finding raises the possibility that dysregulation of Wnt–β-catenin signaling is responsible for the differentiation defects in R595H-Trim71 mutant cells and for the pathology of patients with CH who carry the R608H-TRIM71 mutation (homolog of mouse R595H-Trim71). However, the pathogenic mechanism of the other mutation in Trim71 (R783H-Trim71) needs further investigation.

It is worth mentioning that some of the findings in the study by Liu and colleagues seem to differ slightly from those in prior studies. Liu and colleagues’ findings support gain-of-function mechanisms for CH associated with Trim71 mutations. By contrast, ventriculomegaly and CH-like phenotypes were observed in roughly one-fourth of neuroprogenitor-specific Trim71-KO mice in another study [7]. The low penetrance argues against a unified molecular mechanism of CH. These discrepancies probably indicate that the point mutation in Trim71 (R595H) causes a gain-of-function that affects the Wnt–β-catenin pathway and induces CH, whereas the Trim71-KO caused CH through a different mechanism. There is an interesting parallel with a neurodevelopmental disorder caused by a point mutation in NR2F1. This mutation (R112K) in NR2F1 resulted in a potential gain-of-function pathology due a decrease in the binding activity of NR2F1, which affected the normal function of the Hedgehog pathway [10]. These findings point to the intricacy of developmental neurological disorders and to the heterogeneity of their pathological mechanisms, which warrants further investigation. In particular, to clarify the discrepancies (gain-of-function versus loss-of-function) between the different model systems (point mutations versus knockout), further systematic comparison between mutations in Trim71 (R595H and R783H) and the Trim71-KO are required. Furthermore, whether mechanisms discovered in vitro can be extended to mouse neurogenesis or even to the pathogenesis of human CH remains an open question. Further research using more relevant models, such as animal models or human iPSCs carrying CH-associated mutations would be greatly beneficial to address these issues.

Overall, the study by Liu and colleagues [8] offers a novel perspective on the pathophysiology of CH, which involves a crosstalk between CH-associated genes and developmental signaling pathways. However, diverse types of pathogenesis may underly various point mutations, and aberrant regulation of developmental signaling pathways may only account for a small subset of patients with CH who carry particular mutations (within the 20% of mutation-caused CH cases). The full range of pathological mechanisms resulting from CH-associated mutations still remains to be uncovered. Novel insights from fundamental studies obtained with advanced technologies will broaden our understanding of CH and other neurodevelopmental disorders and may provide new personalized therapeutics to help patients.
